# Seasonal challenges of tropical bats in temperate zones

**DOI:** 10.1038/s41598-022-21076-9

**Published:** 2022-10-07

**Authors:** Maya Weinberg, Omer Mazar, Adi Rachum, Xing Chen, Sophia Goutink, Nora Lifshitz, Rona Winter-Livneh, Gábor Á. Czirják, Yossi Yovel

**Affiliations:** 1 School of Zoology, Faculty of Life Sciences, Tel Aviv University, 6997801 Tel Aviv, Israel; 2 Sagol School of Neuroscience, Tel Aviv University, 6997801 Tel Aviv, Israel; 3 Amutat Atalef, The Israeli Bat Sanctuary (NGO), Beit-Shemesh, Israel; 4 Open Landscape Institute (OLI), The Steinhardt Museum of Natural History, Tel-Aviv University, Tel Aviv, Israel; 5 Department of Wildlife Diseases, Leibniz Institute for Zoo and Wildlife Research, Berlin, Germany; 6 National Research Center for Biodiversity Studies, The Steinhardt Museum of Natural History, Tel-Aviv University, Tel Aviv, Israel

**Keywords:** Animal migration, Biodiversity, Ecological epidemiology, Ecophysiology, Evolutionary ecology, Microbial ecology, Urban ecology, Evolutionary developmental biology, Clinical microbiology, Environmental microbiology, Pathogens, Ecology, Evolution, Microbiology, Zoology, Animal behaviour, Animal physiology

## Abstract

To examine the challenges faced by free-ranging *Rousettus aegyptiacus* living at the northern edge of their distribution, we performed a retrospective analysis of 2196 clinical cases reported by a bat rescue NGO over a period of 36 months, from throughout Israel. All cases of injured bats were evaluated and categorized according to date, place, sex, age, and etiology of the morbidity. The data analysis revealed an increase in all types of morbidity during the wintertime, with more than two-fold the number of cases per week compared to in the summer, over three consecutive years. Moreover, we found that the number of abandoned pups peaked during spring and summer, when adult morbidity is minimal. We characterized two prominent types of previously undescribed morbidities in *R. aegyptiacus*. We also employed GPS tracking to monitor the movement and foraging of dozens of bats, and to examine the potential correlates of elevated winter morbidity. Our results suggest that it is mainly harsh weather that drives the observed winter morbidity, with food limitations playing a minor-role. We hypothesize that *R. aegyptiacus*, of tropical origin, is facing major seasonal survival difficulties near the northern edge of its distribution, probably limiting its spread further northwards still.

## Introduction

Egyptian fruit bats (*Rousettus aegyptiacus*) are highly common across Israel, which is located close to the northernmost edge (Turkey) of their geographic distribution range—i.e., Israel is located within the 90 percentile of their northern population^1,2^ (see Supplementary Fig. 1). Consequently, the bats in Israel must contend with a relatively cold and wet winter in addition to many months of a hot and dry summer. These conditions differ greatly from the tropical conditions of most of the species’ natural range, where it evolved^2,3^.

**Figure 1:**
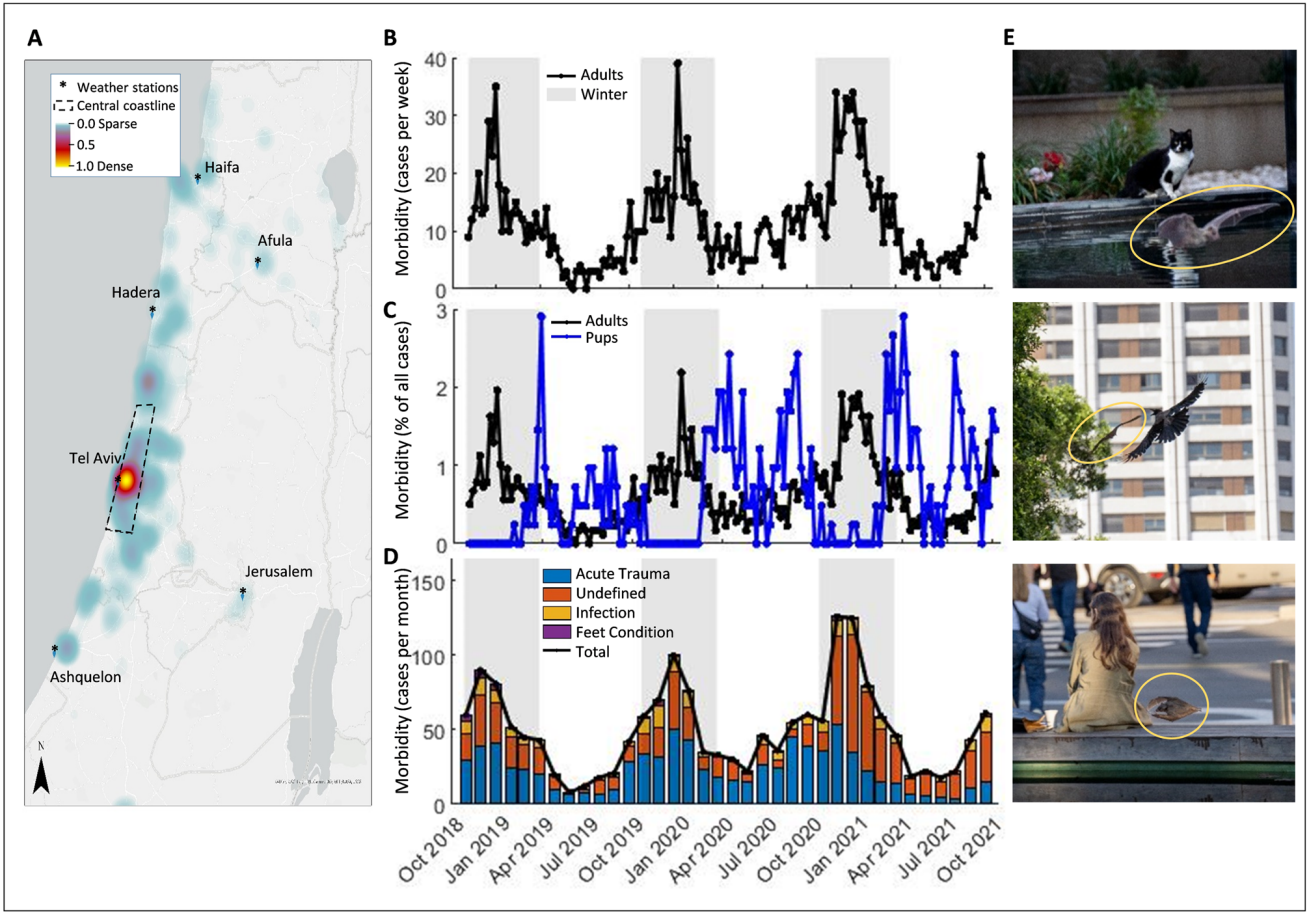
Morbidity of *Roussetus aegyptiacus* in a temperate zone. (**A**) Heat map of all cases (Nov 2018–Oct 2021). The central coastline area is depicted in a black polygon, with the locations of the meteorological stations marked. Note the highest density of cases along the central coastline of Israel. Density values are given as the total number of reported cases per 4 km^2^. Heat-map was generated using ArcGIS^©^ Pro 2.9.3 by ESRI 2021 (URL: https://www.esri.com/en-us/arcgis/products/arcgis-pro/overview). (**B**) The number of adult cases reported during the research period. The graph shows the number of reported cases per week. Gray shaded areas represent the winter periods (for panels **B**–**D**). (**C**) The weekly morbidity percentage of all reported cases for adults (black) and pups (blue). The graph shows a significant negative synchronization between adults and pups morbidity. (**D**) The proportions of morbidity categories for adult cases (acute trauma, infection, feet condition, or undefined). (**E**) *R*. *aegyptiacus* from urban areas (Tel Aviv) encounter various predators and challenges. Top: A bat among people in downtown Tel Aviv, Israel; Middle: A bat threatened by a crow during foraging. Bottom: A bat followed by a stray cat. (Photos courtesy of  Y. Barkai).

It would appear to be challenging for a tropical species to expand its distribution range deep into the temperate zone and *R. aegyptiacus* is the only known fruit bat species to have done so^2,4^. Unlike most temperate bat species, this species does not hibernate^5^ and thus in order to survive it requires a year-round availability of fruit, which is enabled in Israel mostly due to anthropogenic plant cultivation. However, even with sufficient food, the ambient temperature—and especially its drop in winter, presents a substantial barrier for a tropical species^6^; and expanding its geographical boundaries also requires contending with this. Another major challenge for *R. aegyptiacus* in Israel is that of the very high human population density (406 inhabitants per km^2^), which results in frequent interactions between bats and humans. As a species that often roosts in cities, these bats also suffer from common encounters with synanthropic predators such as cats, crows, and rats (see Fig. 1A). Despite these challenges, *R. aegyptiacus* flourishes in urban areas in Israel, mostly due to the widespread availability of deserted structures that it uses as roosting places, and to the agricultural and ornamental fruit trees that provide a year-round supply of fruit^7^.

In light of these challenges, we sought to determine the main causes of morbidity of free-ranging Egyptian fruit bats in Israel. Obtaining reliable morbidity data for wild animals is problematic, and most of the information is based on medical reports from rescue centers, wildlife veterinary hospitals, or governmental laboratories^8–10^. Such data are seldom available for bats. Here, we took advantage of a unique non-governmental organization (NGO) established in Israel in 2016—‘*Amutat Atalef*’. This NGO receives and handles hundreds of reports of bats every year. Each bat case is documented, including the time and location where it was found, as well as the bat’s estimated age and general health condition based on an initial clinical examination. In addition, a picture of the bat is taken in situ. By analyzing 2,196 reported cases arriving over a period of 36 months, we were able to study the main health issues of *R. aegyptiacus* bats in detail, together with their possible explanatory variables. Our results reveal a clear peak in morbidity incidences in winter, mostly related to weather conditions but probably also to some extent to food limitations^11^, highlighting the challenges faced by a bat living far beyond its original tropical range.

## Results

We analyzed a total of 2196 morbidity reports received in Israel between November 2018 and October 2021 (Fig. 1A). The majority of these were for adult bats 1,783 (81.2%), of which 1432 (80.3%) were from urban areas (settlements populated by 30,000 or more people) and the remaining 351 (19.7%) were from rural areas, including small villages, nature reserves, and army bases. Out of all these adult cases, the animal’s sex was identified in only 295 cases (16.6%), with 171 (58%) being females and 124 (42%) being males. 413 (18.8% of all cases) were pups, up to 4 months old, from throughout the country, without sex identification.

We found a dramatic and significant increase in adult bat morbidity during winter (see Fig. 1B) (*p* < 0.0001, F = 90.9, DF = 153, one-way ANOVA). The average number of cases per week during the winter was ~ 2.3 fold higher than that during summer (a mean of 17.0 ± 7.9 SD per week with a maximum of 39 in winter, vs. 7.4 ± 4.6 SD with a maximum of 23 cases in summer). The winter effect was significant for both rural and urban areas (*p* < 0.0001, F = 96, *p* = 0.0.01, F = 10.9, respectively, DF = 153 for both, one-way ANOVA). We also found a significant difference between males and females during the research period, with a higher number of reported cases of female morbidity (*p* = 0.007, *p* < 0.0001 for sex and week, respectively, DF = 307, GLM with Poisson distribution, explained parameter: number of cases, explaining parameters: week number, sex (fixed)).

Pup morbidity exhibited different temporal patterns to those of adults (Fig. 1C, blue line). Pup morbidity peaked twice a year, in April and September, one month after the peak of parturition in March and August^12,13^. Pup morbidity was significantly negatively correlated with adult morbidity (GLM for pup morbidity as the explained parameter and adult morbidity, as the fixed explaining parameter; *p* = 0.0003, F = 13.8, DF = 153).

We observed a significant increase in morbidity in winter of all morbidity types. Of all adult cases, 59.7% were categorized and 40.3% were undefined. Of the diagnosed cases, 78.2% were caused by acute trauma, 18.4% had a putative infectious origin, and 3.4% were attributed to chronic, non-infectious conditions of the animal’s feet. 70% out of the cases with infectious etiology were diagnosed as abscesses of bacterial origin (see “Methods” section for morbidity categories, and Supplementary Fig. 2). The type of morbidity proportions remained constant year-round (*p* = 0.84, 0.66, 0.76, 0.56, for acute trauma, infection, feet condition, and undefined,  respectively, DF = 344, GLM model for the explanatory parameter: rank of cases for morbidity, explanatory parameters: month, see “Methods" section).

**Figure 2:**
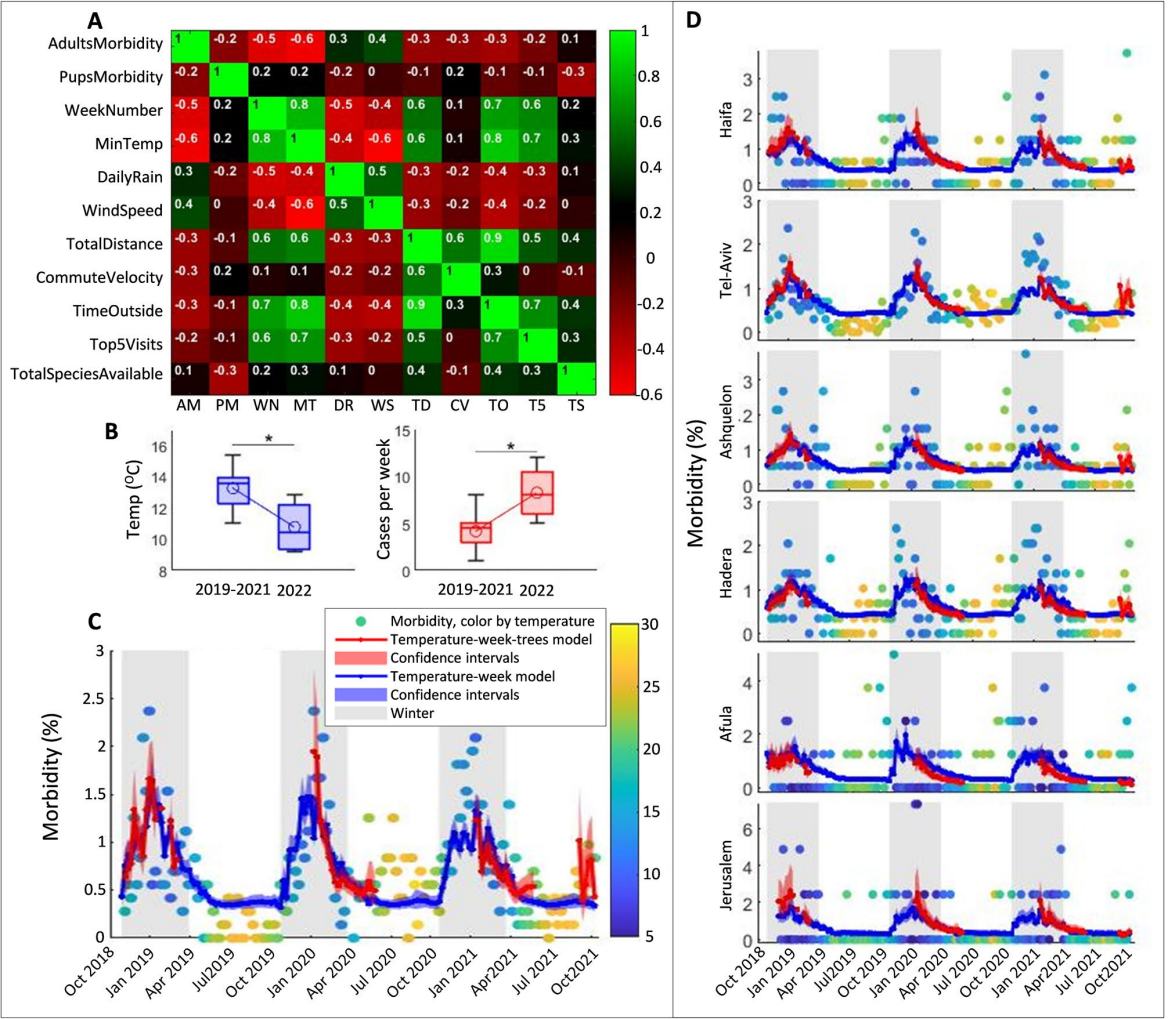
Statistical models explaining morbidity. (**A**) The pairwise correlation matrix between the analyzed variables (only for the central coastline data). Color and text depict the Pearson correlation coefficient (AdultsMorbidity (AM): weekly adult cases; PupMorbidity (PM): weekly pup cases; WeekNumber (WM): week-number from November 1st in each year; MinTemp (MT): average of the daily minimum temperature over a week (°C); DailyRain (DR): the average daily precipitation over a week (mm); WindSpeed (WS): the average of the daily maximal wind-speed (m/s); TotalDistance (TD): the total distance the bats flew each day (km); CommuteVelocity (CV): the average flight velocity during the commute (m/min); TimeOutside (TO): the average time the bats spent outside their colony (hours); Top5Visits (T5): the average number of visits by each bat to one the five preferred tree species; TotalSpeciesAvailable (TS): the average total number of different tree species the bats visited each week). (**B**) Adult Morbidity in March: an extremely cold period in March 2022 (left panel) was correlated with a significant increase in morbidity (right panel) relative to the equivalent period in the previous three years. Asterisks indicate a significant difference, see "Results" section. (**C**) Adult morbidity along the central coastline over time. (**D**) Adults morbidity in six different regions in Israel. In panels (**A**,**D**): The average minimum daily temperature is depicted by a color code ranging from blue (~ 5 °C) to yellow (~ 27 °C). Blue lines and shaded areas indicate the prediction and the 95% confidence intervals of the temperature-winter model for the entire period. Red lines and shaded areas indicate the prediction of the best-fitted model, including the monitored visits to the favorite fruit trees (see text).

Since, compared to the tropics, winter (November–March) in Israel is characterized by relatively harsh weather and a decrease in food (fruit availability), we next examined which of these factors better explained the increase in adult morbidity. For this analysis, only adult cases from the central coastline of Israel were selected (see Fig. 1A, 718 cases, 40.3% of all adult cases). We focused on this region, monitoring the movement and foraging data of 36 bats roosting in a colony at Tel-Aviv University during the same period (2018–2021). First, we examined the pair-wise correlations between all explanatory measured parameters. The time of year, expressed as the week number, was found to be highly correlated with several parameters, (see Fig. 2A, and “Methods” section). We thus tested models with all parameter combinations (see below), and the models’ residuals were also auto-correlated in time (Durbin Watson test, see “Methods” section). We applied a First Order Auto Regression (AR1) model^14^, that predicted the weekly morbidity by the temperature and the previous weekly morbidity. It demonstrated similar performance to the temperature-week model, which included the week along winter as a parameter representing time (R^2^ = 0.36, 0.35; RMSE = 2.8, 2.9, respectively). Therefore, we included in our analysis the week-number as an explanatory parameter with interactions with the other parameters.

Next, we analyzed the effect of weather factors alone on adult morbidity for the entire period (156 weeks) and separately for wintertime only (66 weeks). We examined all possible models with the following weather factors: minimum temperature, daily precipitation, and maximum wind speed; with the week number representing time (with and without interactions between parameters), and ranked them according to their AICs (a total of 23 models for each period, see Sup. Tables 1–3). Temperature and time were significant parameters in all of the top three models. Moreover, the temperature-week model (i.e., a GLM with the minimum temperature, week-number, and their interaction as fixed explanatory parameters) was the only model appearing in the top three models for both periods (*p* < 0.0001, DF = 151, R^2^ = 0.35, GLM for the entire period; *p* < 0.0001, DF = 62, R^2^ = 0.3, GLM for the winter). This model also consisted in the lowest number of coefficients (four), and the AIC difference was insignificant in comparison to the top three models (< 1.45 difference from the best in each period). The significant effects of temperature and time in this model even during the wintertime only (a total of 468 cases in November–March), indicate that the impact of temperature and time was not a result of the winter versus summer batching phenomenon. Consequently, we chose the temperature-week model as the best-fitted model for the weather factors (see Fig. 2C).

We also analyzed which of the model’s parameters better explained morbidity’ and found that temperature explained it better than the week number, see Sup. Table 2, model #11 (temperature only), and model #19 (week only). This is reinforced by the increased morbidity during weeks that were particularly colder than the average (Fig. 2C). For example, in March 2022, the weather in Israel was exceptionally cold, with an average drop of ca. 2.5 °C compared to the average minimum temperature in March 2019–2021 (P = 0.01, F = 8.64, DF = 14, One Way ANOVA). Accordingly, bat morbidity increased significantly (almost doubled) from 4.17 ± 0.6 to 8.25 ± 1 cases per week, Fig. 2C (p = 0.005, F = 11.22, DF = 14, One Way ANOVA).

To determine whether foraging behavior and food availability are significantly related to morbidity, in addition to the weather conditions, we added the data collected by our GPS tags to the temperature-week model (the explanatory factors were all for week averages and comprised total flight distance, commute velocity, time spent outside, number of visits to the top five tree species, total number of tree species visited by the bats, minimum temperature, and week number, see “Methods” section). Again, we examined all possible models (total of 224 models, see “Methods” section) explaining morbidity and chose the best model with the minimum AIC. If several models produced the same AIC, we chose the one with the least number of parameters (see Supplementary Table 4). The model that included temperature, number of visits to the top five tree species, and interaction with the week number (i.e., time) was revealed as the best explanatory model (i.e., the temperature-week-trees model). This is a relatively simple model (with 6 coefficients, including the interactions, all of which are significant), which best explained morbidity (*p* < 0.0001, DF = 62, R^2^ = 0.36 GLM, see Supplementary Table 1). It insignificantly differed from two more complex models (featuring16 and 18 parameters; the delta AIC less than 1.7, see Supplementary Table 4). Compared to the temperature-week model, the new model prominently improved performance: the R^2^ increased from 0.3 to 0.36 and the root mean squared errors decreased from 3.4 to 3.1 (see Fig. 2C and Supplementary Table 1).

The temperature-week-trees model and the temperature-week model for the central coastline were also used to explain adult morbidity countrywide. To this end, the cases were geographically divided according to their closest distance to one of six meteorological stations, and the categorical locations were added to the models as fixed explanatory parameters. Both models explained the morbidity well, see Fig. 2D (the extrapolated temperature-week-trees model: *p* < 0.0001, DF = 354, R^2^ = 0.51, RMSE = 1.97, the extrapolated temperature-week model: *p* < 0.0001, DF = 356, R^2^ = 0.51, RMSE = 2.03, GLM). The marginal effect of the visits to the preferred trees along the central coastline on the countrywide morbidity was still significant (*p*= 0.04, F = 4.05, DF = 354), but with no impact on the R-squared criterion and only a 3% improvement to the RMSE.

## Discussion

Our study sought to elucidate the main drivers and causes of morbidity in free-ranging *R. aegyptiacus* bats and to reveal the challenges these bats face in temperate climates, near the northern edge of their distribution^10^. The study presents a good example of the added value of Citizen Science^15,16^, providing a unique dataset that is extensive and covers a wide temporal and spatial range. Despite the clear benefits, however, certain limitations exist: (1) our ability to analyze a bat’s precise health status is limited since the data provided in the report are often partial. We therefore diagnosed and analyzed only reports providing a clear identification of the examined parameters (such as sex, age, and morbidity cause). In such cases of citizen science, working together with the volunteers and guiding them regarding data collection could have improved our dataset. (2) The data are also biased by human population distribution and behavior. For example, more bats are found in urban than in rural areas (see Fig. 1A). (3) Although, human awareness of bats has been increasing and, therefore, the number of reported cases might also increase, the annual numbers of such reports did not seem to change much during the period of this study (535, 835, and 657 reports annually in 2019, 2020, and 2021, respectively). Despite these limitations, the data collected by the bat-aid NGO remains the most comprehensive information source regarding *R. aegyptiacus* health in Israel and, to our knowledge, is unique for any bat species worldwide. A scheme summarizing the process of exploiting this type of dataset into scientifically valid data and their analysis is provided in Supplementary Fig. S3.

We found that winter is the hardest time of the year for *R. aegyptiacus*, with a strong correlation (Pearson correlation coefficient > 0.4) between the number of reported morbidity cases and all the winter weather parameters we tested (Fig. 2B). During the peak of winter, the number of morbidity cases for adult bats was twice as high as during the summer over the 3 year period (Fig. 1B). Notably, this correlation was also observed within the winter months only suggesting that it does not result from an overall seasonal difference between summer and winter.

Due to their long evolutionary history in relatively equable climates, most tropical species and lineages cannot tolerate the abiotic stresses at higher latitudes, especially cold temperatures and extreme seasonality, and are consequently restricted to the tropics^6^. Novel adaptive traits are thus required in order for tropical species to tolerate stressful abiotic conditions and expand to higher latitudes^17^. A cold winter is known to induce cold stress in a variety of other mammals in terms of growth and production^20^, including a reduction in birth weight and enhanced pre-weaning mortality^21^, lower immunity abilities^22,23^, and a reduction in basic activity at the cellular level^24^. Humans too exhibit higher mortality in the wintertime^25^.

Some bat species living near the northern and southernmost limits of their distribution in the neotropics were found to exhibit stress^18^. Several bat species originating in hot climates undergo torpor upon exposure to cool temperatures^19^. *R. aegyptiacus* does not enter torpor^20,21^. Makin^13^ showed that *R. aegyptiacus* body mass decreases during winter and increases during spring and summer, reaching a peak in October when food availability peaks and the weather is still mild. In addition to the cold temperatures that the bats face during winter, they also face a shortage of fruit, in both abundance and quality. During winter, the bats feed on extremely dry fruit such as Carob (*Ceratonia silique*) and Chinaberry (*Melia azedarach*)^7,22^ which probably affects their energy balance during this period of the year. The reduction in bat body weight reported during winter could also reflect a limited diet. Folivory, for example, was observed mainly during the winter^7^, probably to compensate for nutritional deficits. Our results suggest a significant correlation between higher morbidity and higher foraging efforts, assessed according to the number of visits to the preferred fruit trees. GPS tracking revealed that the bats visited more fruit trees during times of high morbidity. This could reflect a shortage of food, as when trees have less food more trees have to be visited.

Positioned very close to the northernmost edge of the species’ distribution, *R. aegyptiacus* in Israel has to contend with nearly the most extreme winter weather in their distribution range (bats in Turkey and Lebanon might suffer from slightly colder winters, and indeed lower numbers of *R. aegyptiacus* are observed there). This harsh winter (relative to their tropical origin) thus probably limits the distribution range of this species.

Adult *R. aegyptiacus* were found to suffer from three major types of health issues: acute trauma, illnesses of infectious origin, and chronic feet conditions (Sup. Fig. 2). The most common type of morbidity was due to acute trauma (Fig. 1D) with almost 80% of cases belonging to this category. Similar findings, mostly wing trauma, have been reported for other bats (mostly insectivores)^8,23–27^ and other wild species, including birds^9,28^. Among these morbidity types, *R. aegyptiacus* mostly suffer from open fractures and wounds related to encounters with urban predators (crows, cats, dogs, rats) as well as with cars. The second most common group of morbidity was that of infectious etiology. Most cases were due to a bacterial disease with a typical abscess formation in certain typical locations (e.g., around the trapezius muscle in the upper back and neck, the soft tissue around the collar bone, and sometimes in the lumbar area), which coincides with the lesions we often encountered in our semi-captive colony (Weinberg et al. unpublished data). The least common cause of morbidity was that of a chronic feet condition, including any pathology of chronic origin that affects the tarsal and phalanges bones and joints. These lesions manifest in an abnormal stretching of the feet that prevents their normal flexion and consequently their use for suspension. We are familiar with this pathology also in semi-captive bats (Weinberg et al. unpublished data). The two latter causes of morbidity, to the best of our knowledge, have not been previously described in the literature regarding fruit bats and require further investigation. From the biological point of view of infection, bats are mainly examined for viruses^29–31^, despite their also being susceptible to extracellular pathogens such as bacteria^32^ and fungi^33^; however, these pathogens are still relatively poorly studied. Bacterial diseases have been reported in European insectivorous bats^32,34^. Although *R. aegyptiacus* has been identified as a reservoir for *Bartonella*^35^, and several outbreaks of *Yersinia pseudotuberculosis* have been described in captive colonies^36,37^, neither of these has been associated with localized abscesses.

*Rousettus* *aegyptiacus* bats tend to spend less time outside during bad weather conditions (Fig. 2B, Pearson correlation coefficient > 0.8). Despite this, the number of reported cases of all morbidity types increases when the weather is unfavorable, which suggests that those individuals that do forage outside face a higher possibility of harm. Previous studies on bats have described the costs of flying in unfavorable weather conditions, and shown that bats prefer better weather conditions for both flight and migration^38^. A possible explanation for their foraging in bad weather could be that *R. aegyptiacus* must consume food on almost a nightly basis (unlike those bat species that can enter daily torpor or hibernate). It is also likely that the weaker and hungrier individuals must risk foraging under bad weather conditions, and are thus also more prone to harm, including from traumatic injuries.

Furthermore, a previous study found that sick *R. aegyptiacus* refrain from foraging. Following an injection of bacterial lipopolysaccharide (LPS) to imitate a bacterial disease, sick *R. aegyptiacus* “stayed home” and avoided foraging^21^; and, even when they began to forage post-recovery, they did so over a smaller range for shorter periods. It is thus probable that the proportion of ill bats in our sample was lower than their actual occurrence in nature.

The challenges that *R. aegyptiacus* experiences in the Israeli winter are also reflected in their parturition seasonality. Parturition completely stops during winter between November to late February (Fig. 1D), when adult morbidity is at its peak. Only from March onwards did we find lost or abandoned pups, with two clear peaks of incidence in April and September (Fig. 1C), matching the previously reported pup seasons for this species in the region^12,13^. It seems that *R. aegyptiacus* bats in temperate climate regions are barely able to contend with the challenging winter weather conditions and poor fruit availability^12,39^, consequently postponing their parturition period to spring and onwards. Indeed, in our region there are two bat reproduction seasons—towards the beginning and end of winter (i.e., around March and August) in accordance with the weather conditions and fruit availability in the region^12,39^. This explains the negative correlations between pup and adult morbidity. In contrast, in tropical regions, *R. aegyptiacus* has a single annual breeding season^39,40^, with copulation occurring in July–August and parturition in November–December, during the wet summer.

*Rousettus* *aegyptiacus* faces additional challenges in highly populated countries such as Israel. Although more cases of morbidity are reported from urban areas (80% of all reported cases), this finding should be treated with caution due to the reporting bias noted above. No good estimate exists of the differences in fruit bat density between urban and rural regions. As a species that thrives in urban environments, these bats encounter synanthropic predators, including humans (and their vehicles)^41^. We regularly observe cases of cat and dog bites and crow attacks during fieldwork, as well as of bats admitted to medical care. During our study period, 53 cat bite cases and 30 cases of crow attacks were reported (Fig. 1E). Cat bites are known to injure other bat species too^23,42^, which could lead to systemic bacterial infection with *Pasteurella* sp. In anthropogenic surroundings, cats pose such a prominent threat to bats that behavioral changes of avoidance have been shown for the great horseshoe bat (*Rhinolophus ferrumequinum*), mostly during the reproduction season^43^. We also encountered 15 cases of dog bites and 3 cases of vehicle impact. These findings match reports in the literature for other bats^6,42^. The number of fruit bats in urban locations in the region is expected to increase as *R. aegyptiacus* is attracted to cities due to its accessible and versatile diet, the higher temperature in cities, and man-made roosting structures^44–46^. This attraction is probably also occurring in neighboring countries such as Egypt, Jordan, Lebanon, and Turkey, and it might even be happening throughout the entire range of the species^2^. Although *R. aegyptiacus* is a mammal that thrives in the vicinity of humans, direct contact with humans is infrequent. The bats keep a clear distance where possible when sleeping (in caves and deserted buildings, never on rooftops of buildings that are in use) and during foraging (consuming mainly ripe fruit and leaves from trees, and never from the ground or market stalls). *Rousettus* *aegyptiacus* thereby represents a very fragile but successful example of human-wildlife coexistence.

## Methods

The fruit-bat NGO ‘*Amutat Atalef*’ received ~ 700 reports every year between 2018 and 2021. These reports are conveyed through the “WhatsApp” application, to a group of volunteers who rush to assist the bats. Between November 2018 and October 2021, every text message sent through this group was integrated into a table, which was organized as follows: date, time, location, rural versus urban surroundings, sex, estimated age, clinical description of the morbidity, and/or mortality. All these entries were compared to the provided images and were evaluated blindly and independently by two examiners (MW, a veterinarian doctor specializing in bats for over a decade; and SG, a student trained by MW), who are well acquainted with *R. aegyptiacus*. A total of 2196 cases were recorded and analyzed between 4/11/2018 and 23/10/2021. Cases reported without noting the location were excluded from the analysis. There is currently no accurate way to determine a bat’s age visually, so the bats were divided into two age categories: adults or young pups, which were always less than 4 months old, based on visual parameters as described in^13,47^.

Morbidity was categorized into four major types of health issues: *acute trauma*, *infection*, *feet condition,* and *undefined* (Supplementary Fig. 2). Morbidity refers to adult bats only, since pups, when found, were all categorized as lost, e.g., involuntarily separated from their mothers. Note that at this age pups are still dependent on their mothers for supplementary food, thermoregulation, and protection during the day^48^. Acute trauma included any type of injury such as wing fracture, cat bite, crow peck, vehicle impact, and all other types of recent wounds. *Infection* included all situations of a clear ongoing inflammatory and infectious process, such as swollen joints and manifestation of abscesses with no clear trauma. *Feet condition* referred to a chronic health condition resulting from an abnormal extension of the tarsal and metatarsal bones that prevented the bats from hanging, and would eventually lead to exhaustion and death. *Undefined* referred to all cases without sufficient information to enable identification as belonging to one of the main categories, or to some other condition. Cases of mortality were similarly reported, through the WhatsApp group. Animals were treated according to the nature of the problem, and some of them died later without notification among the group.

We collected climate data from the archive of the national meteorological database service (https://ims.data.gov.il/) for the relevant period. Information on the daily minimum and maximum temperature and precipitation was collected from the same database. Wind speed was collected according to the hour and averaged per day. For weekly analysis, the daily wind speed data were averaged over the relevant period.

To track fruit availability and consumption by the bats during wintertime, as well as the amount of time spent outside the roost for commuting and foraging, a total of 36 bats (19 females and 17 males) were tracked using GPS. Bats were tracked for a total duration of 62 weeks during the following periods: 11/2018- 03/2019 five bats, 01/2020–05/2020 15 bats, 01/2021–05/2021 12 bats, and 09/2021–11/2021 four bats, all in the Tel Aviv area^49^. The fruit trees visited by the bats, including their location and species identification, were verified by visiting the actual locations where the bats foraged.

### Data analysis

Morbidity was defined as the number of cases reported each week or month. Because it is a countable parameter, we used the Generalized Linear Model (GLM) with the Poisson distribution. The differences between the cases in winter and summer were tested by One-Way ANOVA. Winter was defined as the period between 1/11 and 31/03. We divided the data into rural and urban cases and repeated the analysis for each group separately. The effect of sex on morbidity throughout the year was analyzed using the GLM, with morbidity set as the explanatory parameter and the week-number from the start of the winter (i.e., 1–52, for each year) and the reported sex as the explanatory parameters.

To determine whether the proportions of the morbidity types changed over the course of the year, we clustered the cases into months and ranked them. A GLM model for the ranked morbidity as a function of the month number in the year (i.e., 1–12, for the research period of 26 months) was then executed and analyzed.

For a better understanding of the influence of the weather, foraging behavior, and time on adult morbidity, we first executed a cross-correlation test between all of the potential explanatory parameters and found them to be highly correlated. We, therefore, included the interactions between the parameters in the analyzed models. Because the foraging data were collected only during limited periods, we also tested the correlations between pairs of factors in the relevant intersecting times for each pair. Next, we analyzed the auto-correlations of the residuals of the models in time using the Durbin Watson test. The residuals were auto-correlated and, consequently, the time was explicitly added to the models as the week number from the start of winter. Next, we generated GLM models for the morbidity with all possible combinations of these parameters as the explanatory factors (with and without interactions) for the entire research period, wintertime only, and the period of the collected tracking data. The best model was selected according to the minimal AICC (Akaike’s Information Criterion Corrected), maximum R^2^, and minimal coefficients’ number (see Sup. Tables 1–3).

To analyze the influence of the bats' foraging behavior and food availability on morbidity, we calculated the weekly average of the following parameters from each tracked bat: time spent outside the roosting colony (hours), maximum flight velocity during the commute for each night (m/minute), the total number of different fruit tree species visited by the bats each week (normalized by the number of tracked bats in that week, to obtain a ‘per-bat’ value), and the number of visits to the five tree species preferred by these bats (normalized per bat as above). The five most visited species were as follows: *Ficus microcarpa, Ficus ribiginosa, Melia azedarach, Eucalyptus camaldulensis,* and *Washingtonia robusta*.

All analyses were performed using MATLAB^©^ R2021b. Figure number 1A was generated using ArcGIS^©^ Pro 2.9.3.

## Supplementary Information

Supplementary Information.

## Data Availability

The datasets used and analyzed during the current study are available from the corresponding author upon request.
