# Supplementary material for: Seasonal challenges of tropical bats in temperate zones
Source: Sci Rep. 2022 Oct 7;12:16869. doi: 10.1038/s41598-022-21076-9 (PMC9546901; doi:10.1038/s41598-022-21076-9)
Supplement: Supplementary file 1 — Supplementary Information. [file 41598_2022_21076_MOESM1_ESM.pdf]

Supplementary: Seasonal challenges of tropical bats in temperate zones

Seasonal challenges of tropical bats in temperate zones

Maya Weinberg*†[1,3], Omer Mazar†[2], Adi Rachum[1], Xing Chen[1], Sophia Goutink[1], Nora Lifshitz[3], Rona Winter-Livneh[4], Gábor Á. Czirják[5], Yossi Yovel[1,2,6]

Supplementary Figure 1

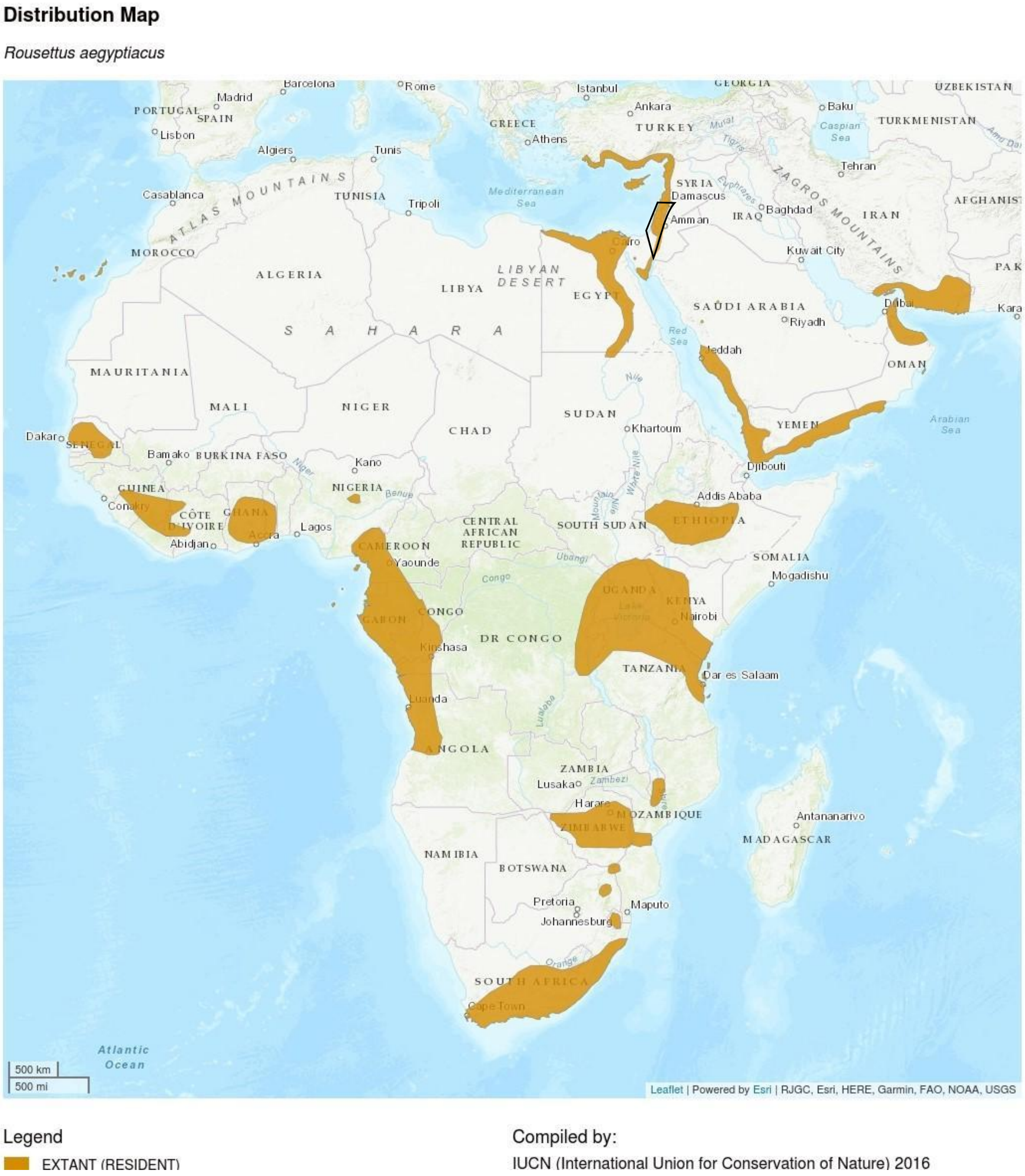

Supplementary Figure S1*: Distribution map of R.aegyptiacus.* Origin with permission: IUCN (2016) https://www.iucnredlist.org/fr/species/29730/22043105. Israel depicted in black polygon.

Supplementary Figure 2

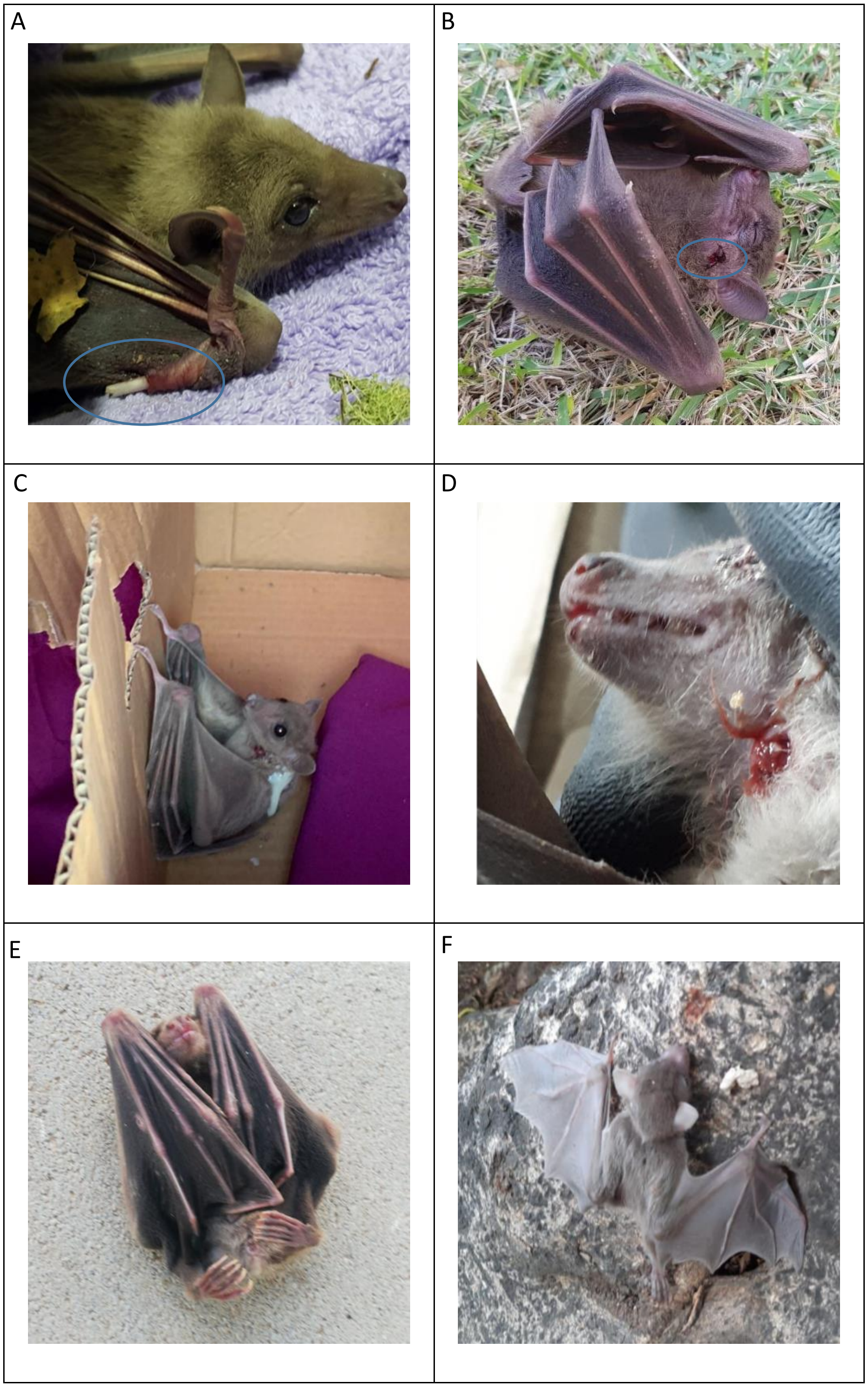

Supplementary Figure S2: **Different types of morbidities** as identified from pictures. **(A)** An open fracture is classified as acute trauma. (**B)** Pecking by a crow is classified as acute trauma. (**C)** Pus from a cervical abscess is classified as an infectious disease. **(D)** Cervical abscess classified as infectious disease **(E)** Feet condition **(F)** Lost pup. All pictures courtesy of the Israel Bat Sanctuary.

Supplementary Figure 3

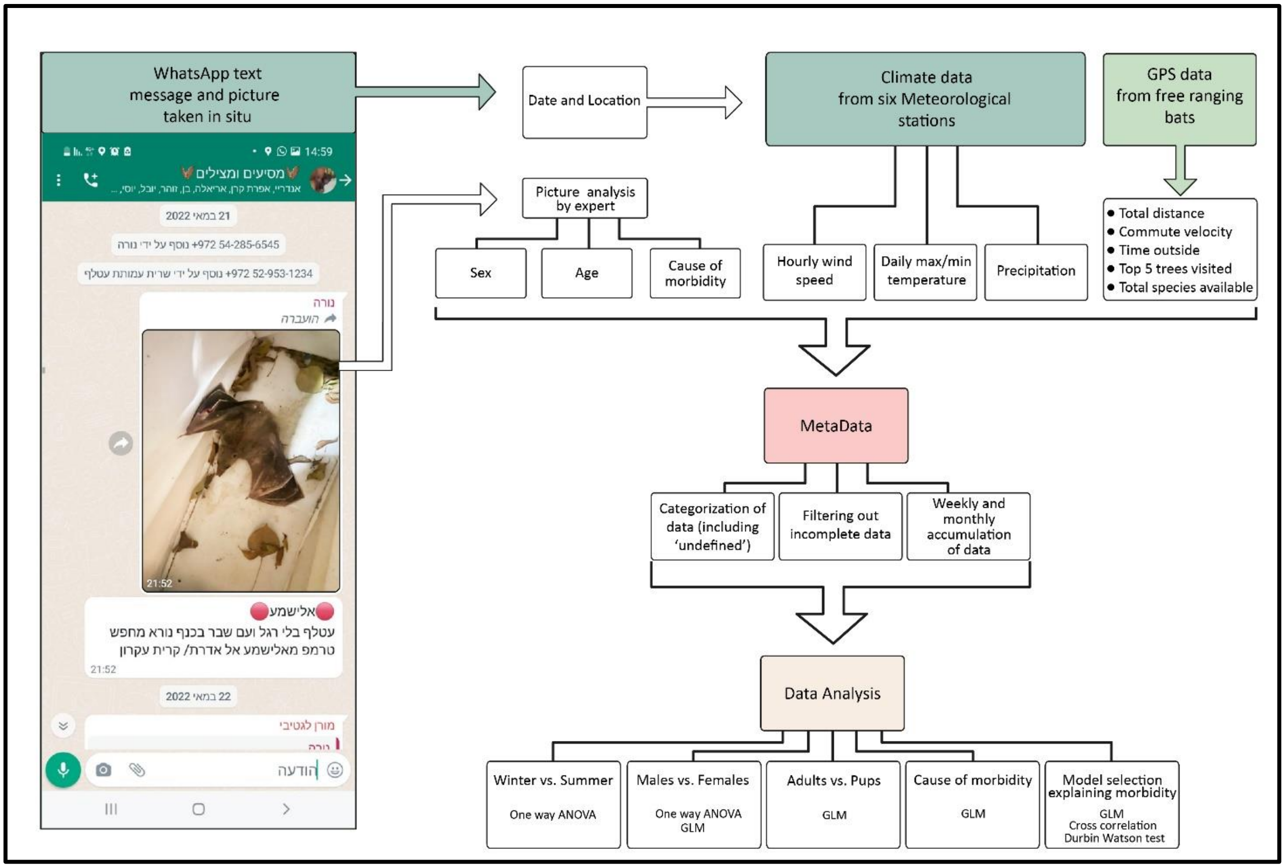

Supplementary Figure S3: Flow chart transforming data collected in the field by citizen-scientists into valid scientific data. The first phase of this process involves the first phase is collecting data from the given text message with a picture; as some data is (such as date and location or climate data), while other data may require expert analysis (such as estimation of age, sex, and cause of morbidity). The second phase involves creating a valid metadata table, including categorization, accumulation, and data filtering. The third phase involves data analysis, including a set of statistical tests pertaining to the analyzed parameter. In the final phase, we select the best explanatory model for our results.

Supplementary Table 1

Model name	Period	Region	Estimate	N	R^2	AIC	RMSE	Coeff p-vals
Temperature-week	Full period	Central coastline	Y=4.44- 0.16X1 -0.08X2 + 0.004(X2:X1)	155	0.35	350	2.88	X1: <0.00001<br>X2: <0.00001<br>X1:X2: <0.00001
Temperature-week	Winter	Central coastline	Y=5.68- 0.24X1 -0.23X2 + 0.014(X2:X1)	66	0.3	108	3.45	X1: <0.00001<br>X2: 0.0003<br>X1:X2: 0.0014
Temperature-week	Tracking time	Central coastline	Y=5.2- 0.2X1 -0.14X2 + 0.007(X2:X1)	62	0.3	129	3.37	X1: <0.00001<br>X2: <0.00001<br>X1:X2: <0.00001
Best-fitted	Tracking time	Central coastline	Y=5.2- 0.25X1 -0.17X2 + +0.02X3 + 0.01(X2:X1) – 0.0007(X3:X2)	62	0.36	121	3.11	X1: <0.00001<br>X2: <0.00001<br>X3: 0.0007<br>X1:X2: <0.00001<br>X3:X2: 0.0015
Temperature-week	Full Period	Countrywide	Y=2.27 - 0.13X1 - 0.07X2 0.003(X1:X2) + $b^{\dagger}$X4	155	0.46	3140	1.88	X1: <0.00001<br>X2: <0.00001<br>X1:X2: <0.00001
Temperature-week	Tracking time	Countrywide	Y=3.26 - 0.18X1 - 0.14X2 + 0.008X3 + 0.006(X1:X2) + b^{*}X4	62	0.51	1202	2.03	X1: <0.00001<br>X2: <0.00001<br>X1:X2: <0.00001<br>X4: <0.00001
Best-fitted	Tracking time	Countrywide	Y=3.28 - 0.2X1 - 0.12X2 + 0.008(X1:X2) - 0.0003(X3:X2) + b^{*}X4	62	0.51	1217	1.97	X1: <0.00001<br>X2: <0.00001<br>X3: 0.04<br>X1:X2: <0.00001<br>X3:X2: 0.01<br>X4: <0.00001

Table S1: **The performance of the selected models for the central coastline and countrywide for the entire period of research (4/11/2018 to 23/10/2021); wintertime only (1/11 to 31/3 annually); and during the period that the bats were tracked** (see Fig. 2C). Coefficients: Y-adult morbidity; X1- minimum temperature; X2-week-number; X3- number of visits to the five most favored tree species; X4 –categorical location of the report; b†: coefficients of the categorical locations are not shown in the table. N- The number of weeks for each model. AIC- The Akaike Information Criterion, RMSE- Root Mean Squared Error. Coeff p-vals: The p-values of the estimated coefficients in each model.

Supplementary Table2

#	formula	nCoeff	weeks	R^2	AICc	Δ AICc	RMSE	p-Val	DF
1	adult_counts ~ 1 +(min_temp_C * wind_speed_ms)* week_from_winter	8	155	0.41	350.62	0	2.77	1.48E-23	147
2	adult_counts ~ 1 +(min_temp_C + wind_speed_ms)* week_from_winter	6	155	0.38	350.67	0.05	2.85	1.44E-24	149
3	adult_counts ~ 1 +(min_temp_C)* week_from_winter	4	155	0.35	352.06	1.44	2.88	1.02E-25	151
4	adult_counts ~ 1 +(min_temp_C + daily_rain_mm + wind_speed_ms)* week_from_winter	8	155	0.38	354.22	3.6	2.85	2.84E-23	147
5	adult_counts ~ 1 +(min_temp_C + daily_rain_mm)* week_from_winter	6	155	0.35	356.19	5.57	2.87	3.48E-24	149
6	adult_counts ~ 1 +(min_temp_C * daily_rain_mm)* week_from_winter	8	155	0.36	359.26	8.64	2.85	4.59E-23	147
7	adult_counts ~ 1 +(min_temp_C * daily_rain_mm * wind_speed_ms)* week_from_winter	16	155	0.43	360.46	9.84	2.67	3.04E-20	139
8	adult_counts ~ 1 +min_temp_C + wind_speed_ms	3	155	0.33	370.45	19.83	3.05	1.32E-23	152
9	adult_counts ~ 1 +min_temp_C + daily_rain_mm + wind_speed_ms	4	155	0.33	372.72	22.1	3.04	6.76E-23	151
10	adult_counts ~ 1 +min_temp_C * wind_speed_ms	4	155	0.36	373.09	22.47	3.02	2.91E-22	151
11	adult_counts ~ 1 +min_temp_C	2	155	0.33	373.49	22.87	3.06	5.79E-24	153
12	adult_counts ~ 1 +min_temp_C + daily_rain_mm	3	155	0.33	374.34	23.72	3.04	2.27E-23	152
13	adult_counts ~ 1 +(wind_speed_ms)* week_from_winter	4	155	0.33	374.39	23.77	3.09	3.81E-22	151
14	adult_counts ~ 1 +(daily_rain_mm * wind_speed_ms)* week_from_winter	8	155	0.35	375.6	24.98	3.01	6.22E-20	147
15	adult_counts ~ 1 +(daily_rain_mm + wind_speed_ms)* week_from_winter	6	155	0.33	376.13	25.51	3.06	6.32E-21	149
16	adult_counts ~ 1 +min_temp_C * daily_rain_mm	4	155	0.33	376.63	26.01	3.04	2.04E-22	151
17	adult_counts ~ 1 +min_temp_C * daily_rain_mm * wind_speed_ms	8	155	0.36	380.13	29.51	3.01	5.43E-20	147
18	adult_counts ~ 1 +(daily_rain_mm)* week_from_winter	4	155	0.3	388.9	38.28	3.11	1.01E-20	151
19	adult_counts ~ 1 + week_from_winter	2	155	0.27	396.11	45.49	3.23	5.44E-21	153
20	adult_counts ~ 1 +daily_rain_mm * wind_speed_ms	4	155	0.19	433.14	82.52	3.35	1.33E-17	151
21	adult_counts ~ 1 +daily_rain_mm + wind_speed_ms	3	155	0.15	441.14	90.52	3.42	1.49E-17	152
22	adult_counts ~ 1 +wind_speed_ms	2	155	0.15	443.18	92.56	3.45	2.06E-17	153
23	adult_counts ~ 1 +daily_rain_mm	2	155	0.07	482.5	131.88	3.6	5.44E-12	153

Table S2: Analysis of all possible GLM models explaining morbidity along the coastline for the entire research period, with weather factors and week number as explaining factors. (Green) We selected model #3 (i.e., the temperature-week model) as the best-fitted model because it has the lowest number of coefficients, with no difference in the AIC criteria (Δ AICc = 1.4 <4) compared to models #1, and #2.

Moreover, this model is the only model that well explains the morbidity both year-round and during wintertime only (see Supplementary Table 2). In this model, the explanatory fixed variables are the minimum temperature and the first week following the start of winter, including their interactions. Yellow rows - the two models with lower AICc's during the full period are depicted also in Supplementary Table 2. Orange rows - models with low AIC during wintertime only. The joint analysis of the entire period and the wintertime indicates that wind speed and precipitation have only a marginal effect on morbidity. The total number of reported cases used to fit these models was 718. **Table columns**: **formula –** the models' specifications described by Wilkinson Notation**, nCoeff –** number of coefficients (i.e. explanatory factors, including their interactions), **weeks –** weeks included in the model, **R^2 –** the percentage of 'variance explained', **AICc -** Akaike's Information Criterion Corrected, **Δ AICc-** the difference between each model's AICC and the minimal AICc (model #1), **RMSE-** root mean squared error**, p-Val** – the model's p-Value**, DF-** Degrees of Freedom.

Supplementary Table 3

#	formula	nCoeff	weeks	R^2	AICc	Δ AICc	RMSE	p-Val	DF
**1**	adult_counts ~ 1 +(min_temp_C * daily_rain_mm)* week_from_winter	8	66	0.4	107.28	0	3.31	1.57E-05	58
2	adult_counts ~ 1 +(min_temp_C)* week_from_winter	4	66	0.3	108.71	1.43	3.45	2.92E-06	62
3	adult_counts ~ 1 +(min_temp_C + daily_rain_mm)* week_from_winter	6	66	0.32	110.95	3.67	3.38	1.11E-05	60
4	adult_counts ~ 1 +(min_temp_C * wind_speed_ms)* week_from_winter	8	66	0.4	111.36	4.08	3.35	5.79E-05	58
5	adult_counts ~ 1 +(min_temp_C + wind_speed_ms)* week_from_winter	6	66	0.3	113.09	5.81	3.46	2.38E-05	60
6	adult_counts ~ 1 +(min_temp_C + daily_rain_mm + wind_speed_ms)* week_from_winter	8	66	0.32	113.54	6.26	3.37	3.95E-05	58
7	adult_counts ~ 1 +(min_temp_C * daily_rain_mm * wind_speed_ms)* week_from_winter	16	66	0.5	120.47	13.19	3.09	0.0003	50
8	adult_counts ~ 1 +(daily_rain_mm * wind_speed_ms)* week_from_winter	8	66	0.23	122.91	15.63	3.54	0.0005	58
9	adult_counts ~ 1 +(daily_rain_mm + wind_speed_ms)* week_from_winter	6	66	0.17	127.18	19.9	3.65	0.0009	60
10	adult_counts ~ 1 +min_temp_C	2	66	0.11	129.63	22.35	3.78	0.0005	64
11	adult_counts ~ 1 +min_temp_C * wind_speed_ms	4	66	0.14	130.49	23.21	3.75	0.003	62
12	adult_counts ~ 1 +min_temp_C + wind_speed_ms	3	66	0.11	130.8	23.52	3.77	0.001	63
13	adult_counts ~ 1 +min_temp_C + daily_rain_mm	3	66	0.11	131.93	24.65	3.77	0.001	63
14	adult_counts ~ 1 +min_temp_C + daily_rain_mm + wind_speed_ms	4	66	0.11	133.49	26.21	3.77	0.003	62
15	adult_counts ~ 1 +min_temp_C * daily_rain_mm	4	66	0.11	134.26	26.98	3.77	0.004	62
16	adult_counts ~ 1 +(wind_speed_ms)* week_from_winter	4	66	0.08	134.5	27.22	3.82	0.006	62
17	adult_counts ~ 1 +wind_speed_ms	2	66	0.04	137.15	29.87	3.88	0.01	64
18	adult_counts ~ 1 +(daily_rain_mm)* week_from_winter	4	66	0.07	138.23	30.95	3.84	0.02	62
19	adult_counts ~ 1 +min_temp_C * daily_rain_mm * wind_speed_ms	8	66	0.16	139.59	32.31	3.72	0.02	58
20	adult_counts ~ 1 +daily_rain_mm + wind_speed_ms	3	66	0.05	139.69	32.41	3.87	0.03	63
21	adult_counts ~ 1 +daily_rain_mm * wind_speed_ms	4	66	0.05	141.1	33.82	3.87	0.07	62
22	adult_counts ~ 1 + week_from_winter	2	66	0.01	142.58	35.3	3.95	0.3	64
23	adult_counts ~ 1 + daily_rain_mm	2	66	0.01	143.26	35.98	3.93	0.15	64

Table S3: Analysis of all possible GLM models explaining morbidity along the coastline during wintertime only, incorporating weather factors and week number. As in Table S1, but here we analyzed wintertime only (between 1/11 and 31/03 across three years). The temperature-week model is indicated in green as the best-fitted model for morbidity. Green row - the temperature-week model. Yellow rows -

models with low AICs for the entire period (see Supplementary Table 1). Orange rows - models with low AICcs (*Δ AICc <4)* during the wintertime. The total number of reported cases used to fit these models was 468. **Table columns:** as in Table S2.

Supplementary Table 4

#	formula	nCoeff	weeks	R^2	AICc	Δ AICc	RMSE	p-Val	DF
1	adult_counts ~ 1 +(TotalDistance * MeanTimeOutside * min_temp_C)* week_from_winter	16	62	0.57	120.95	0	2.31	2.28E-07	46
2	adult_counts ~ 1 +(CommuteVelocity + MeanTimeOutside + Vistis_Top5TreesNorm)* week_from_winter	8	62	0.39	121.21	0.26	3.09	5.55E-08	54
3	adult_counts ~ 1 +(Vistis_Top5TreesNorm + min_temp_C)* week_from_winter	6	62	0.36	122.63	1.68	3.11	1.09E-08	56
4	adult_counts ~ 1 +(MeanTimeOutside + Vistis_Top5TreesNorm + min_temp_C)* week_from_winter	8	62	0.37	122.87	1.92	3.09	4.89E-08	54
5	adult_counts ~ 1 +(MeanTimeOutside + Vistis_Top5TreesNorm)* week_from_winter	6	62	0.34	122.95	2	3.16	2.35E-08	56
6	adult_counts ~ 1 +(CommuteVelocity + MeanTimeOutside + Vistis_Top5TreesNorm + species_availbleNorm)* week_from_winter	10	62	0.43	123.18	2.23	3.02	2.54E-07	52
7	adult_counts ~ 1 +(MeanTimeOutside * species_availbleNorm * min_temp_C)* week_from_winter	16	62	0.59	123.46	2.51	2.55	8.86E-07	46
8	adult_counts ~ 1 +(CommuteVelocity + Vistis_Top5TreesNorm + min_temp_C)* week_from_winter	8	62	0.38	123.64	2.69	3.1	6.20E-08	54
9	adult_counts ~ 1 +(CommuteVelocity + MeanTimeOutside + Vistis_Top5TreesNorm + min_temp_C)* week_from_winter	10	62	0.41	123.94	2.99	3.05	2.20E-07	52
10	adult_counts ~ 1 +(MeanTimeOutside + Vistis_Top5TreesNorm + species_availbleNorm + min_temp_C)* week_from_winter	10	62	0.42	124	3.05	3.03	2.19E-07	52
11	adult_counts ~ 1 +(TotalDistance + CommuteVelocity + MeanTimeOutside + Vistis_Top5TreesNorm)* week_from_winter	10	62	0.44	124.14	3.19	2.97	1.77E-07	52
12	adult_counts ~ 1 +(TotalDistance + Vistis_Top5TreesNorm + min_temp_C)* week_from_winter	8	62	0.37	124.32	3.37	3.1	6.08E-08	54
13	adult_counts ~ 1 +(TotalDistance + CommuteVelocity + Vistis_Top5TreesNorm + min_temp_C)* week_from_winter	10	62	0.42	124.44	3.49	3.04	2.12E-07	52
14	adult_counts ~ 1 +(MeanTimeOutside + Vistis_Top5TreesNorm + species_availbleNorm)* week_from_winter	8	62	0.41	124.46	3.51	3.08	1.50E-07	54
15	adult_counts ~ 1 +(Vistis_Top5TreesNorm + species_availbleNorm + min_temp_C)* week_from_winter	8	62	0.39	125.84	4.89	3.06	8.67E-08	54
16	adult_counts ~ 1 +(TotalDistance + CommuteVelocity + MeanTimeOutside + Vistis_Top5TreesNorm + species_availbleNorm)* week_from_winter	12	62	0.47	126.37	5.42	2.87	4.08E-07	50
17	adult_counts ~ 1 +(MeanTimeOutside * species_availbleNorm)* week_from_winter	8	62	0.38	126.57	5.62	3.24	1.77E-07	54
18	adult_counts ~ 1 +(MeanTimeOutside * Vistis_Top5TreesNorm * min_temp_C)* week_from_winter	16	62	0.55	126.83	5.88	2.51	8.12E-07	46
19	adult_counts ~ 1 +(Vistis_Top5TreesNorm * min_temp_C)* week_from_winter	8	62	0.36	126.99	6.04	3.12	9.02E-08	54
20	adult_counts ~ 1 +(CommuteVelocity + min_temp_C)* week_from_winter	6	62	0.33	127.24	6.29	3.33	1.07E-07	56

21	adult_counts ~ 1 +(TotalDistance + Vistis_Top5TreesNorm + species_availbleNorm + min_temp_C)* week_from_winter	10	62	0.41	127.47	6.52	3.06	3.61E-07	52
22	adult_counts ~ 1 +(MeanTimeOutside * Vistis_Top5TreesNorm)* week_from_winter	8	62	0.35	127.55	6.6	3.15	1.46E-07	54
23	adult_counts ~ 1 +(TotalDistance + MeanTimeOutside + Vistis_Top5TreesNorm + species_availbleNorm)* week_from_winter	10	62	0.42	127.6	6.65	3.03	5.20E-07	52
24	adult_counts ~ 1 +(CommuteVelocity + MeanTimeOutside + Vistis_Top5TreesNorm + species_availbleNorm + min_temp_C)* week_from_winter	12	62	0.44	127.6	6.65	3	8.08E-07	50
25	adult_counts ~ 1 +(TotalDistance + MeanTimeOutside + Vistis_Top5TreesNorm)* week_from_winter	8	62	0.35	127.82	6.87	3.12	1.37E-07	54
26	adult_counts ~ 1 +(CommuteVelocity + Vistis_Top5TreesNorm + species_availbleNorm + min_temp_C)* week_from_winter	10	62	0.41	127.83	6.88	3.05	3.48E-07	52
28	adult_counts ~ 1 +(TotalDistance + CommuteVelocity + Vistis_Top5TreesNorm)* week_from_winter	8	62	0.37	128.07	7.12	3.21	3.52E-07	54
29	adult_counts ~ 1 +(TotalDistance + CommuteVelocity + MeanTimeOutside + Vistis_Top5TreesNorm + min_temp_C)* week_from_winter	12	62	0.44	128.09	7.14	2.96	5.30E-07	50
30	adult_counts ~ 1 +(MeanTimeOutside + species_availbleNorm)* week_from_winter	6	62	0.34	128.28	7.33	3.33	1.99E-07	56
31	adult_counts ~ 1 +(MeanTimeOutside + species_availbleNorm + min_temp_C)* week_from_winter	8	62	0.37	128.28	7.33	3.29	4.43E-07	54
32	adult_counts ~ 1 +(CommuteVelocity * min_temp_C)* week_from_winter	8	62	0.37	128.45	7.5	3.25	5.19E-07	54
33	adult_counts ~ 1 +(CommuteVelocity + MeanTimeOutside)* week_from_winter	6	62	0.32	128.55	7.6	3.35	1.33E-07	56
34	adult_counts ~ 1 +(TotalDistance + MeanTimeOutside + Vistis_Top5TreesNorm + min_temp_C)* week_from_winter	10	62	0.37	128.79	7.84	3.07	2.49E-07	52
35	adult_counts ~ 1 +(CommuteVelocity + MeanTimeOutside + species_availbleNorm)* week_from_winter	8	62	0.37	128.95	8	3.27	5.34E-07	54
36	adult_counts ~ 1 +(CommuteVelocity * MeanTimeOutside)* week_from_winter	8	62	0.35	129.47	8.52	3.3	7.08E-07	54
37	adult_counts ~ 1 +(TotalDistance + MeanTimeOutside + Vistis_Top5TreesNorm + species_availbleNorm + min_temp_C)* week_from_winter	12	62	0.44	129.55	8.6	2.97	7.39E-07	50
38	adult_counts ~ 1 +(CommuteVelocity + species_availbleNorm + min_temp_C)* week_from_winter	8	62	0.37	129.61	8.66	3.27	4.72E-07	54
39	adult_counts ~ 1 +(TotalDistance + CommuteVelocity + min_temp_C)* week_from_winter	8	62	0.37	129.91	8.96	3.29	6.11E-07	54
40	adult_counts ~ 1 +(min_temp_C)* week_from_winter	4	62	0.3	130	9.05	3.37	3.31E-08	58

Table S4: Analysis of all possible GLM models explaining morbidity along the coastline during the monitoring period with temperature, tracking data, and week number as explanatory factors. The table shows the top 40 models out of all possible models (237) sorted according to the AICc criterion. Green row shows model #3 was selected as the best-fitted model because it has the lowest number of coefficients with no difference in the AIC criteria (Δ AICc = 1.4 <4) in comparison to the models in lines 1-2. This best-fitted model explains morbidity by minimum temperature, the number of visits to the top 5 tree species, and the

interaction with week number (see Results). Model #2 presents very similar results but with higher complexity (i.e., more parameters) Blue row - Model #40 is the temperature-week model. 36 bats were tracked during the following periods: 11/2018–03/2019 five bats, 01/2020–05/2020 15 bats, 01/2021–05/2021 12 bats, and 09/2021–11/2021 four bats in the Tel Aviv area. During these periods 362 cases were reported. Table columns: as in Table S2.
